# Psychiatric Goals of Care at the End of Life: A Qualitative Analysis of Medical Records at a Geriatric Psychiatric Outpatient Clinic

**DOI:** 10.1155/jare/2104985

**Published:** 2024-11-29

**Authors:** Helena Kullenberg, Gert Helgesson, Niklas Juth, Anna Lindblad

**Affiliations:** ^1^Department of Health Promoting Science, Sophiahemmet University, Stockholm, Sweden; ^2^Stockholm Centre for Healthcare Ethics (CHE), Department of Learning, Informatics, Management and Ethics (LIME), Karolinska Institutet, Solna, Sweden; ^3^Centre for Research Ethics and Bioethics (CRB), Department of Public Health and Caring Sciences, Uppsala University, Uppsala, Sweden

**Keywords:** care goals, end-of-life, geriatric psychiatry, goals of care, palliative, qualitative

## Abstract

**Objectives:** Person-centered care emphasizes patient choice and autonomy and is considered an important means for improving the quality of care and quality of life for older adults with multiple chronic conditions and functional limitations. In implementing person-centered care, goals of care based on the patient's preferences are considered fundamental. Psychiatry is generally practiced in a curative paradigm, and little is known about the goals of care in geriatric psychiatric settings. In this study, goals of care as documented in care plans and medical records in geriatric psychiatric outpatient care have been explored, with a special focus on the end of life.

**Methods:** This study was based on a descriptive qualitative content analysis of medical records of patients enrolled at an outpatient clinic for geriatric psychiatry at the time of death. It was complemented by a basic quantitative analysis of patient characteristics.

**Results:** A total of 66 medical records were included, with a male/female ratio of 41/59% and a mean age of 83 years (66–104 years). Among psychiatric diagnoses, depression predominated. The dataset was generally limited, and clearly defined goals of care were sparsely presented. Therefore, the included medical records were analyzed twice: first regarding goals of care and second regarding patient wishes and requests. In both cases, the highest level of abstraction in terms of themes was achieved. Analysis of goals of care resulted in the themes patient well-being and care arrangements. Analysis of patient wishes resulted in the themes active patienthood and living and being.

**Conclusion:** Goals of care were often disease-oriented, focusing on recovery or symptom management, whereas analysis of patients' wishes revealed personal goals other than remission, including outspoken existential needs. The results call for further research on the interplay between person-centered care and the goal-planning process and point to the potential of a palliative approach in geriatric psychiatric care involving patients with complex comorbidities and multilevel needs.

**Summary**

• Goals of care are an important means for implementing person-centered care, but in the present study, the goals were often disease-oriented, focusing on symptom management or recovery.
• Patient wishes documented in medical records reveal goals other than remission, including outspoken existential needs.
• There is a need to discuss how person-centered goals of care can be formulated in geriatric psychiatry.

## 1. Introduction

Person-centered care is a medical approach that emphasizes patient choice and autonomy [1]. It involves transitioning from the traditional disease-focused biomedical model for medicine, where the patient is a receiver of medical measures, to a model where the patient is actively involved in decision-making [2]. Although relevant in all aspects of healthcare, person-centered care is considered especially important in the care of older adults with multiple chronic conditions and functional limitations as a means for improving quality of care and quality of life [1].

Patients with complex health problems and limitations are frequent in geriatric psychiatry, a medical subspecialty for patients over 65 years of age suffering from mental health disorders. Physical comorbidity and unmet psychological and social needs are common factors that may negatively influence well-being and capacity to lead an autonomous life [3]. A multidisciplinary approach to assessment, diagnosis, and treatment is considered state of the art, and collaboration with general practitioners, social services, and hospital physicians is essential [3–6].

Several factors are essential to implement person-centered care. Among these, goals of care, based on the patient's preferences and documented in a goal-oriented care plan, are considered fundamental [7]. Goals of care may, at least ideally, be understood as “desired health expectations that are formulated through the thoughtful interaction between a human being seeking medical care and the healthcare team in the healthcare system and are appropriate, agreed on, documented, and communicated” [8]. Based on patient values and priorities, goals of care may guide medical decisions [9, 10]) and support motivation and outcomes for service users [11]. Furthermore, to be effective, the goals of care must be reassessed regularly [8]. This means that the care plan is to be considered a dynamic document and, as such, an important tool for evaluation and planning [12].

Psychiatry is generally practiced in a curative paradigm [13]. Little is known about the goals of care in geriatric psychiatric settings, or at least studies from this particular subspeciality seem to be lacking. In the authors' clinical experience, goals of care in geriatric psychiatry tend to focus on remission even in situations beyond cure, and psychiatric palliative care approaches are rarely outspoken. Therefore, in this explorative, qualitative study, we have aimed to analyze the goals of care as documented in care plans and medical records in geriatric psychiatric outpatient care. Our focus has been the end of life, based on the assumption that this is a phase where care goals may be discussed and reviewed, possibly opening an adjustment from a curative to a palliative approach.

## 2. Methods and Materials

### 2.1. Design

This is a one-center, exploratory study. It is based on a qualitative content analysis of a consecutive selection of medical records of patients enrolled at an outpatient clinic for geriatric psychiatry at the time of death, complemented by a basic quantitative analysis of patient characteristics.

### 2.2. Setting

The study was conducted at the largest outpatient clinic for geriatric psychiatry in Stockholm, Sweden. The clinic cares for patients aged +65 years with psychiatric conditions, apart from primary neurocognitive disorders.

### 2.3. Data Collection

Medical records documented in the e-health system Take Care within 3 months before the patient's death were identified using the Take Care database Intelligence, with the following inclusion criteria:

• The patient had at least one visit to the outpatient clinic from January 1, 2013, to 31 October, 2017.
• The patient was deceased before 31 October, 2017.
• Medical records contained at least one record documented at the Department for Geriatric Psychiatry (outpatient or inpatient) within 3 months before the time of death.
• The patient had ongoing contact with the outpatient clinic at the time of death.

Medical records of patients whose enrollment was terminated before the time of death were excluded. Patients who were not formally enrolled at the polyclinic, for instance, because their only visit had been limited to a consultative visit at a geriatric ward without any further intervention from the department, were also excluded.

All identified medical records were reviewed regarding inclusion and exclusion criteria in the first step. In the second step, study-specific case report forms on patient characteristics (e.g., sex, age, and diagnosis) were filled to facilitate the quantitative analysis.

### 2.4. Data Analysis

The textual data included free-text medical records (documented within 3 months before the patient's death) and standardized care plans (documented within 12 months before the patient's death) from the geriatric psychiatric clinic.

All processing and analysis were performed with coded data. A study-specific code number replaced patient names and personal identification numbers, and the names of healthcare professionals were deleted. The code list was stored separately from the extracted data and was only accessible to the researchers directly involved in the study. The results have been presented as themes and categories without the possibility of identifying individual patients or healthcare professionals. Citations used to illustrate themes and categories have been presented so that confidentiality has been ensured.

### 2.5. Quantitative Analysis

Data were registered and analyzed using Epi Info version 6 and Epi Info Analysis version 3.3.2. Descriptive statistical methods were used for basic quantitative analysis of included and excluded data. Excluded medical records were analyzed regarding age, sex, and the reason for exclusion. Included records were analyzed regarding age, sex, psychiatric diagnosis, and physical problems.

### 2.6. Qualitative Analysis

The qualitative analysis was led by AL in close collaboration with HK and continuously discussed with GH and NJ. The authors' preunderstanding is based on their professional backgrounds: AL as a senior physician in psychiatry and HK as a psychiatric nurse, both at the time of the study working at the Department of Geriatric Psychiatry at Norra Stockholms Psykiatri. The coauthors NJ and GH contributed to the analysis from their point of view as philosophers and medical ethicists.

**Figure 1:**
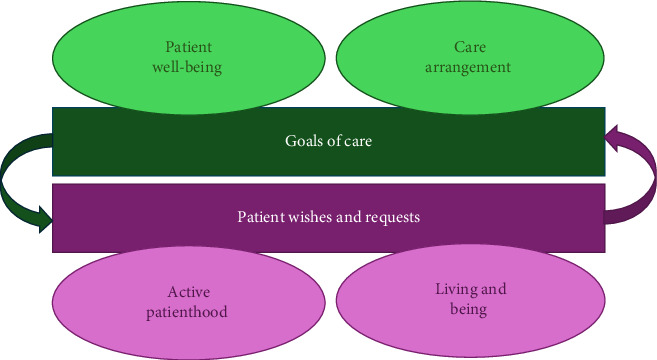
Data were analyzed twice, first regarding goals of care and second regarding patients' wishes and requests. In both cases, the highest level of abstraction in terms of a theme was achieved.

Textual data from the included medical records were analyzed with descriptive qualitative content analysis using the terminology suggested by Graneheim and Lundman [14]. Due to the explorative nature of the study, an inductive approach without preset categories was chosen. Data were read several times, and content connected to the research question was marked as meaning units, condensed, and coded. Explicit goals of care turned out to be very scarce. However, the data contained information on patients' wishes, and it was decided to add this aspect to the analysis. Thus, the included medical records were analyzed twice: first regarding goals of care and second regarding patient wishes and requests. In both cases, the highest level of abstraction in terms of a theme was achieved (see Figure 1). Authors AL and HK read all patient records separately and then coded and categorized the findings in collaboration. All authors discussed the final analysis until a consensus was reached.

### 2.7. Ethical Considerations

This study was reviewed and approved by the Regional Ethical Review Board in Stockholm, Dnr 2017/2046-31/2, and by the caregiver SLSO, permit 18-260. Only historical medical records of deceased patients were included, and no informed consent could therefore be obtained. GDPR does not apply to deceased persons. Nevertheless, confidentiality must be ensured to maintain trust in the medical services and research institutions. In the current study, all data were pseudonymized, and all processing and analysis were performed with coded data. A study-specific code number replaced patient names and personal identification numbers. The names of healthcare professionals were deleted. The code list was stored separately from the extracted data and was only accessible to the researchers directly involved in the study.

## 3. Results

In total, 174 medical records were obtained from patients deceased within three months of the last contact or visit at the outpatient clinic. After being reviewed for inclusion and exclusion criteria, 66 medical records remained.

### 3.1. Patient Characteristics

The male/female ratio was 41/59%, and the mean age was 83, ranging between 66 and 104 years. Among psychiatric diagnoses, depression predominated (44%). Physical comorbidity was common: in 56 of 66 included medical records, at least one physical symptom, diagnosis, or disability was mentioned, and in 80% of these cases (*n* = 45), more than one physical problem occurred.

#### 3.1.1. Contact With Outpatient Clinic Before Death

In 71% of the included medical records, standardized care plans had been formulated within 1 year before the patient's death. Visits or telephone contacts with the patients themselves or others involved in their care (e.g., family, house doctors, and nurses) had taken place within 1 month before death in more than 55% of cases. Six patients had been subject to inpatient psychiatric care within 3 months before their death.

### 3.2. Excluded Records

In total, 108 medical records were excluded. A majority of these related to patients who had undergone psychiatric evaluation by the consultation service from the Department of Geriatric Psychiatry while admitted to a geriatric clinic, i.e., there had been no further follow-up at the outpatient clinic for geriatric psychiatry.

The male/female ratio among excluded records was 42/58%, and the mean age was 84, ranging between 64 and 97 years.

### 3.3. Goals of Care

The dataset was generally limited, and clearly defined goals of care were sparsely presented. Nevertheless, the qualitative analysis of the manifest content resulted in two overarching themes: patient well-being and care arrangements (see Table 1). These themes are descriptive, illuminating “the red thread” of the data and thus related to the research question [15].

#### 3.3.1. Patient Well-Being

The first theme, patient well-being, describes how goals may be oriented toward the patient as a person. Three categories were included: symptom-oriented, pharmaceutical, and life-oriented goals.

#### 3.3.2. Symptom-Oriented Goals of Care

This category describes goals that focus on the aim of psychiatric treatment in terms of psychiatric symptoms and disease progression. Some goals aim at partial, others at complete symptom relief. However, how symptoms should be measured and the treatment evaluated was not mentioned anywhere. Also, some goals of care did not focus on present symptoms but on preventing future symptoms, such as new affective episodes. Sometimes, the goals of care were not aimed at symptom intensity but at the patient's capacity to cope with the symptoms.

*“[…] Goal: become free from anxiety and depression*.*“ (*care plan in medical record nr 1)

*“[…] Problem formulation: Staff at the patient's nursing home experience difficulties in meeting the patient's need for care, perceive the patient as very anxious. Goal: to reduce patient anxiety.”* (care plan in medical record nr 32)

*“[…] Goal: Continued stable mental health with stable mood.”* (care plan in medical record nr 2)

*“[…] Goal: prevent depression and exacerbation.”* (care plan in medical record nr 53)

#### 3.3.3. Pharmaceutical Goals of Care

This category entails goals focusing on the pharmacological treatment itself rather than the effects of it. It includes goals of care explicitly stating a limitation of addictive drugs such as benzodiazepines. The goals did not entail any concrete alternative strategies for the patient, i.e., what to do instead of using medication. Neither was it mentioned what effects a goal like this might have on the patient. Sometimes, a discussion about medication was included as a goal of care or at least an interim goal.

“[...]. Goal: Continue to taper Oxascand.” (care plan in medical record nr 167)

“[...]. Goal: If possible, make the anxiety more manageable by taking prescribed medication, i.e., Voxra, Abilify, and Oxascand, and continued contact with the geriatric psychiatric outpatient clinic. Subgoal: Try not to increase the consumption of Sobril and Stesolid.” (care plan in medical record nr 113)

“[…] Subgoal: Free from depressive symptoms. Discussion about antidepressant medication. Home visit.” (care plan in medical record nr 84)

### 3.4. Life-Oriented Goals of Care

This category describes goals of care that focus on aspects outside the biomedical model, thus representing a more holistic view of the patient. Some goals addressed existential needs, such as reflection upon existential matters and meaningful activities. Other goals focused on strengthening the patient's strategies to cope with symptoms and life challenges to improve quality of life.

*“ [...]. Goal: Find a balance between effort and rest. Find good strategies to use against anxiety escalation.”* (care plan in medical record nr 167)

“Agrees that the purpose of the supportive counseling should be the possibility to express existential thoughts and to be supported in finding activities or actions that can improve the patient's quality of life” (free text in medical record nr 118)

“[…] Goal: Find strategies to deal with the worry/anxiety and thus improve quality of life” (care plan in medical record nr 51)

#### 3.4.1. Care Arrangement

The second theme, care arrangement, covered two categories: “goals associated with care setting” and “goals concerning change of care focus.” In both instances, goals pointed out the direction of the patient's further journey through the healthcare system.

#### 3.4.2. Goals of Care Associated With Care Setting

In some medical records, goals stated where the patient's psychiatric symptoms would best be cared for.

“[...] Goal: The short-term goal at present is that the patient is cared for as an inpatient as the situation at home is untenable despite increased efforts from the home care service.” (care plan in medical record nr 129)

“[…] Goal: Receive a dementia diagnosis and a guardian. Try to manage her situation at the outpatient clinic. Subgoal: Continued support to manage her life.” (care plan in medical records nr 128)

#### 3.4.3. Goals of Care Concerning Change of Care Focus

This category involves goals of care where a shift of care focus was proposed or decided. In some instances, patients were severely ill with somatic disorders, and palliative care was the only option left. This was not stated in the psychiatric care plan but was outspoken in plain-text medical records. Some medical records entailed statements about futility, i.e., situations where further psychiatric treatment was refrained from. These tended to occur in records of patients undoubtedly at the end of life.

“Depressive episode, assessed in remission after the summer. At present, treatment of the addiction problem is a priority” (free text in medical record nr 171)

“The patient mainly needs palliative care, and we agree that it is not relevant to send him to the psychiatric emergency room for possible admission to a psychiatric clinic.” (free text in medical record nr 111)

“Do not assess the patient as depressed, and there is no evidence of acute psychosis. Rather, this is a case of incipient dementia development in a very old and severely somatically ill woman. At present, I cannot see any indication for any psychopharmaceutical treatment, and the patient cannot undergo a memory assessment.” (free text in medical record nr 63)

“Bed-bound uremia patient with lifelong anxiety and many years of overconsumption of sedatives. Little room for psychiatric interventions.” (free text in medical record nr 141)

### 3.5. Patient Wishes and Requests

Although clearly stated goals of care were scarce in the material, patient records revealed much about patients' requests and wishes. This analysis yielded two themes: active patienthood and living and being (see Table 1). Again, the themes are descriptive and correlate to the original research question [15].

#### 3.5.1. Active Patienthood

The first theme, active patienthood, covers four categories and describes what patients wanted in their role as patients, i.e., requests about what they wanted from their psychiatric care rather than personal life goals.

#### 3.5.2. Symptom Management

Some patients expressed wishes for specific measures or treatments to cope with symptoms.

“Expresses a wish to reduce the newly added medications that were introduced during the last treatment session. Feels that she has a lot of side effects from these, as described above.” (free text in medical record nr 51)

#### 3.5.3. Prevent Relapse

This category describes patient wishes associated with preventing relapse in their psychiatric disorder.

“The patient acknowledges the need for counseling. Says he notices faster changes in his mood towards the depressive side. Is vigilant about his early symptoms of relapse.” (free text in medical record nr 171)

##### 3.5.3.1. Medical Assessment

This category describes how patients have requested medical assessment due to somatic symptoms and where psychiatric healthcare professionals are involved.

“The patient desires to have the dizziness and stomach problems examined, which have not been examined by the general practitioner. We have tried to refer the patient to Geriatrics for inpatient assessment, but Geriatrics rejected the referral. We are now writing a referral to the patient's GP for a decision on further investigation of the patient's dizziness, possibly an ear/nose/throat assessment and gastroscopy.” (free text in medical record nr 41)

### 3.6. Refrain From Treatment

This category describes how patients have decided to refrain from further psychiatric treatment, inpatient care, or other medical interventions.

“The patient then states that he wishes to stop Mirtazapine as his liver function has deteriorated and that he has been told that he ‘will die'.” (free text in medical record nr 118)

“Ongoing depression. Does not want further medical treatment for this. Does not want to be hospitalized. Can consider trying a supportive contact with a nurse during home visits. (free text in medical record nr 144)

### 3.7. Living and Being

The second theme, captured as “living and being,” covers two categories focusing on what patients wanted for themselves outside their patient role, i.e., wishes and requests associated with them as persons and not as carriers of symptoms.

#### 3.7.1. Existential Needs

Medical records entailed information on existential needs expressed by the patients, such as a wish to talk about specific topics or to perform a particular activity.

“Feels some hope for the future and specifies that he wants help to round up his life and accept his life situation, which he hopes to achieve through counseling.” (free text in medical records nr 126)

#### 3.7.2. Improved Quality of Life

This category includes concrete examples of what patients would like to do or would like to happen to feel better. Compared to the goals of care in the structured care plans, these wishes can easily be evaluated by asking whether *X* or *Y* has happened or taken place.

“The patient wishes to start exercising so that he can walk with a cane and not be dependent on a walker.” (free text in medical record nr 144)

“Been active in writing and reading a lot, attended the writing course. Would like to start something like that again.” (free text in medical record nr 154)

## 4. Discussion

The main finding of this study was the lack of person-centered, evaluable goals in care plans and plain-text medical records. Patients' values and priorities were not documented, and although most of the included medical records entailed standardized care plans, these were often nonspecific and sweeping in their formulations. The focus was mainly on recovery or symptom management from a medical view, hence lacking a person-oriented perspective. Seldom were goals formulated as interventions or activities to be carried out by healthcare professionals, whereas the role of the patient was left uncommented, i.e., more in line with the biomedical model for healthcare than with a person-centered care perspective [16].

This is by no means a unique finding for geriatric psychiatry; that patient values and priorities are overlooked or left out has been shown previously in other healthcare settings [10, 17]. In addition, a recent study on goals of care forms in a sample of older patients in Quebec showed that 67% of the included medical charts entailed the goals of care form, but only 16% of these were completed [18]. When it comes to mental health services, a systematic integrative review by Stewart et al. [11] concluded that although goal planning is regarded as an important process promoting shared decision-making and supporting motivation for change in people suffering from mental disorders, there is no golden standard for how the process is to be conducted. Furthermore, goals formulated by healthcare professionals tended to focus mainly on symptom control, whereas patients also included aspects relevant to daily life in their goals [11]. Similar findings have been reported from research on self-defined serious illness goals [19]. Hence, these results are in line with our analysis of patient wishes, which showed that patients addressed matters beyond symptoms and pharmacology, including existential issues, suggesting that further collaborative work between the patient, healthcare professional, and, in some instances, next of kin could have resulted in more specific, person-oriented and achievable goals of care. We therefore assume that person-centered interactions did take place at least to some extent, but results were not documented as goals of care, or the process stopped halfway; the patient's narrative was present but not incorporated in the medical decision-making process and planning of care [2, 20].

Not including patients' wishes and goals in care plans is problematic since it affects the care focus and possibly leads to discordance between healthcare professionals and patients [21]. If limited to handling symptoms and treatment options, quality of life and well-being aspects may be missed, and modifiable factors producing ill-health remain unaddressed. This may indicate a need for further education and training of healthcare professionals in geriatric psychiatry to achieve a more person-centered goal-planning process [11], as well as further research on knowledge translation and involvement of patients in the goal-planning process in geriatric psychiatry.

In cases of severe persistent mental illness (SPMI), remission may not be achievable, and consideration of patients' priorities besides symptom control may be especially important to consider. Here, a goal-planning process may lead to a switch from a curative to a palliative care approach where the patient's whole situation and well-being are considered within a palliative care framework [6, 22, 23].

The concept of palliative psychiatry has gained increasing interest in the past years [23–27], and a definition has been proposed by Trachsel et al. [23]. Based on the WHO definition of palliative care, the proposal outlines how psychiatric care for people with SPMI could focus on quality-of-life goals by addressing multilevel needs [23]. Research has also shown that psychiatrists identify a need for a palliative approach in certain cases of SPMI [28, 29]. Furthermore, there is evidence that patients with SPMI can engage in reasoning about end-of-life planning similarly to patients without SPMI [30]. Although the idea of a palliative care approach is not limited to geriatric psychiatry, it may be relevant for older adults with complex morbidities and limitations suffering from mental disorders. Nevertheless, in the present study of goals of care at the end of life, structured conversations involving a discussion of palliative care are scarce. Whether this means that such conversations have not taken place or if they simply have not been documented in the medical records is not possible to determine.

### 4.1. Different Goals at the End of Life?

At the outset of this study, we wanted to explore psychiatric goals of care at the end of life. We chose a geriatric psychiatric setting since this patient population is often in a vulnerable position, with a combination of advanced age and psychological, psychiatric, and physical needs [3]. However, the analysis revealed that although death, dying, and palliative care were sometimes mentioned in plain-text medical records, an outspoken shift of goals of care from a curative to a palliative approach was rare. In some cases, this may be explained by the patient dying unexpectedly. On the other hand, one could argue that the mere combination of advanced age, somatic comorbidities, and psychiatric disorders would be enough for a care plan aiming at increased quality of life rather than just symptom reduction or even cure.

Our analysis revealed that decisions about a shift of care focus were made in some instances, but neither psychiatric healthcare professionals nor patients saw psychiatric care as part of the patient's palliative care; healthcare professionals suggested ending psychiatric care for palliative care to take place, and patients asked to refrain from psychiatric treatment due to life-threatening disorders. In addition, none of these decisions were followed by an outspoken psychiatric palliative approach. Although palliative psychiatry is a novel concept, we do find it somewhat surprising that the notion of palliative care was lacking even in cases where patients clearly were at the end of life.

Changing the focus to palliative care means that the goal of care is no longer to cure but to relieve suffering and achieve the best possible quality of life [23]. In a psychiatric setting, determination of prognosis is difficult (to say the least), and the concept of futility is not yet sufficiently understood [31–33]. In addition, the word “palliative” itself may be problematic as it is often associated with death and dying [24, 26]. This may partly explain the lack of outspoken palliative goals in our data but gives no clue to the absence of person-centered goals.

A means to support the focus on quality of life, and life content taken quite literally, is to use one or several conversation tools to help patients explore their goals, hopes, and desires regarding life at the end of life (a.k.a. advance care planning). One example is the *Go Wish* cards or some national version thereof (such as the *DöBra* cards adjusted to the Swedish context) for conversations on preferences and values in the context of care toward the end of life [34]. An obvious advantage of such conversation support is that the cards contain specific ideas of what might be particularly important for the person with limited time left. The card content varies from hands-on issues such as “being free of pain,” “getting my finances in order,” doing specific things, or meeting dear friends one last time, to focusing on existential or religious matters. There are also some *wild cards*, where things not found in the deck can be added. By using the cards as a starting point for conversation, patients can be guided to identify issues of particular importance to them. The cards can help patients raise issues that would otherwise be difficult to address and encourage them to express their personal goals outside the medical context. With specific goals and desires listed, it will be easier for caregivers to support realizing these goals.

### 4.2. Strengths and Limitations

This is a small explorative study on a topic that, as far as we know, has not been addressed scientifically before. The fact that the data were derived from a single clinic limits the transferability of the results. Nevertheless, the results open important questions on the interplay between person-centeredness and goals of care in geriatric psychiatry, suggesting room for further research. Here, a multicenter approach could be of value to gain more insight into the practice of establishing care goals and how the process is perceived by the patients and healthcare professionals involved. However, this may not be easily achieved in a Swedish setting since geriatric psychiatry in Sweden is still fragmented and underdeveloped [35]. Therefore, a feasible multicenter approach in Sweden must probably include other psychiatric settings as well, for instance by applying a mixed-methods approach that includes perspectives of both caregivers and service users. From the review by Stewart et al. [11], we know that there is a need for further research into the effectiveness of different goal-setting interventions and such evaluations should, if possible, be integrated into any future projects.

An apparent weakness of the study is that all data originate from medical records, i.e., text produced by healthcare professionals without the direct involvement of the patient. This means that only a selection of all that has been said in the appointments between patients and professionals has been available for analysis. Previous research has indicated that patients' preferences, feelings, and beliefs about their condition generally are not documented in medical records [2], and it is possible that patients have expressed other wishes, requests, or goals that have been filtered out during the act of documenting.

Furthermore, the data have offered a particular challenge. As Graneheim, Lindgren, and Lundman [15] point out, it is important to avoid “surface descriptions and general summaries” when conducting inductive content analysis [15]. The condensed language of the medical records and the scarcity of care goals have kept the results on a descriptive level corresponding to the research question but limiting the possibilities of abstraction.

Medical records have multiple purposes. Besides providing documentation of the patient's care, they serve as communication tools between caregivers in a team or between different disciplines, provide evidence in the case of complaints, and constitute a legal and ethical obligation, to mention a few [36]. Therefore, there is a risk that patients and healthcare professionals may assess the importance of certain information differently. On the other hand, goals of care are to be formulated in cooperation and documented in medical records, so if not found there, where else?

Over the past years, there has been a shift toward a more explicit focus on care plans and goals of care in psychiatric care in Sweden. It is possible that medical records documented today have a more person-centered approach than the data we have presented here. However, until this point, our clinical experience has not given us reason to believe so.

## 5. Conclusion

This study reveals a lack of personalized, evaluable goals of care in the medical records of patients enrolled in geriatric outpatient psychiatry at the end of life. When present, goals of care were often disease-oriented, focusing on medical recovery or symptom management. However, analysis of patients' wishes revealed personal goals other than remission, including outspoken existential needs.

The results call for further research on the interplay between person-centered care and goal planning and point at the potential of a palliative approach in geriatric psychiatric care involving patients with complex comorbidities and/or severe persistent mental disorders.

## Figures and Tables

**Table 1:** Qualitative content analysis regarding goals of care and patients' wishes and requests.

	Subcategory	Category	Theme
Goals of care	Symptom relief	Symptom-related goals of care	Patient well-being
	Maintaining psychiatric well-being		
	Limit the use of addictive drugs	Pharmaceutical goals of care	
	Pharmaceutical discussion		
	Existential conversations	Life-oriented goals of care	
	Strategy for improving quality of life		
	Inpatient care	Goals of care associated with care setting	Care arrangement
	Outpatient care		
	Focus on addiction	Goals of care concerning change of care focus	
	Focus on social planning at the end of life		
	Focus on palliative care		
	Refrain from treatment at the end of life		

Patients' wishes and requests	Supportive counseling	Symptom management	Active patienthood
	More frequent visits		
	Decrease side effects		
	Medical treatment for symptom reduction		
	Preventive counseling	Prevent relapse	
	Continue medical treatment		
	Somatic examination	Medical assessment	
	Somatic consultation		
	Declines hospital care	Refrain from treatment	
	Declines medical treatment		
	Declines medical interventions		
	Existential conversations	Existential needs	Living and being
	Conversations about loneliness		
	Conversations about self-esteem		
	To find explanations		
	Catching up before death		
	Improve physical capacity	Improved quality of life	
	To be more active		
	Improve economic situation		
	Resume meaningful activities		

*Note:* The table displays subcategories, categories, and themes for each analysis.

## Data Availability

The medical records used to support the findings of this study have not been made available since the data concern sensitive personal information. They cannot be shared without pseudonymization. Researchers interested in these data are encouraged to contact the main author for inquiries.

## References

[1] Kogan A. C., Wilber K., Mosqueda L. (2016). Person-Centered Care for Older Adults With Chronic Conditions and Functional Impairment—A Systematic Literature Review. *Journal of the American Geriatrics Society*.

[2] Ekman I., Swedberg K., Taft C. (2011). Person-Centered Care—Ready for Prime Time. European Journal of Cardiovascular Nursing. *Journal of the Working Group on Cardiovascular Nursing of the European Society of Cardiology*.

[3] World Health Organization & World Psychiatric Association (1997). Psychiatry of the Elderly—A Consensus Statement. *Aging & Mental Health*.

[4] Gustafson L. (2000). Elderly Psychiatry in an International Perspective [Äldrepsykiatri I Ett Internationellt Perspektiv]. *Lakartidningen*.

[5] Wattis J. (1996). What an Old Age Psychiatrist Does. *BMJ*.

[6] World Health Organization & World Psychiatric Association (1998). Organization of Care in Psychiatry of the Elderly—A Technical Consensus Statement. *Aging & Mental Health*.

[7] American Geriatric Society (2016). Person-Centered Care—A Definition and Essential Elements. *Journal of the American Geriatrics Society*.

[8] Stanek S. (2017). Goals of Care—A Concept Clarification. *Journal of Advanced Nursing*.

[9] Boardman J., Dave S. (2020). Person-Centred Care and Psychiatry: Some Key Perspectives. *BJPsych International*.

[10] Secunda K., Wirpsa M. J., Neely K. J. (2020). Use and Meaning of “Goals of Care” in the Healthcare Literature: A Systematic Review and Qualitative Discourse Analysis. *Journal of General Internal Medicine*.

[11] Stewart V., McMillan S. S., Hu J. (2022). Goal Planning in Mental Health Service Delivery: A Systematic Integrative Review. *Frontiers in Psychiatry*.

[12] Menear M., Girard A., Dugas M., Gervais M., Gilbert M., Gagnon M. P. (2022). Personalized Care Planning and Shared Decision Making in Collaborative Care Programs for Depression and Anxiety Disorders: A Systematic Review. *PLoS One*.

[13] McKellar D., Ng F., Chur-Hansen A. (2016). Is Death Our Business? Philosophical Conflicts over the End-of-Life in Old Age Psychiatry. *Aging & Mental Health*.

[14] Graneheim U. H., Lundman B. (2004). Qualitative Content Analysis in Nursing Research-Concepts, Procedures, and Measures to Achieve Trustworthiness. *Nurse Education Today*.

[15] Graneheim U. H., Lindgren B. M., Lundman B. (2017). Methodological Challenges in Qualitative Content Analysis: A Discussion Paper. *Nurse Education Today*.

[16] Farre A., Rapley T. (2017). The New Old (And Old New) Medical Model—Four Decades Navigating the Biomedical and Psychosocial Understandings of Health and Illness. *Healthcare*.

[17] Wihlborg C., Rasmussen B., Fürst C.-J. (2023). Undignified End-Of-Life Care—the Reality of Palliative Care?. *Läkartidningen*.

[18] Plaisance A., Morin M., Turcotte S., Laflamme B., Heyland D. K., Leblanc A. (2023). A Quality Assessment of Goals of Care Forms in a Sample of Older Patients in Various Care Settings in Quebec, Canada. *Cureus*.

[19] Schellinger S. E., Anderson E. W., Frazer M. S., Cain C. L. (2018). Patient Self-Defined Goals: Essentials of Person-Centered Care for Serious Illness. *American Journal of Hospice and Palliative Care*.

[20] Hedberg C. (2020). Patient-Centered Consultation-Good for Both Patient and Physician [Patientcentrerad Konsultation-Bra För Både Patient Och Läkare]. *Lakartidningen*.

[21] Klement A., Marks S. (2020). The Pitfalls of Utilizing “Goals of Care” as a Clinical Buzz Phase—A Case Study and Proposed Solution. *Palliative Medicine Reports*.

[22] Radbruch L., De Lima L., Knaul F. (2020). Redefining Palliative Care—A New Consensus-Based Definition. *Journal of Pain Symptom Management*.

[23] Trachsel M., Irwin S. A., Biller-Andorno N., Hoff P., Riese F. (2016). Palliative Psychiatry for Severe and Persistent Mental Illness. *The Lancet Psychiatry*.

[24] Gieselmann A., Vollmann J. (2020). A Palliative Concept for Psychiatry? Conceptual Considerations on the Advantages and Limitations of Closer Cooperation Between Palliative Care and Psychiatry. *Nervenarzt*.

[25] Lindblad A., Helgesson G., Sjöstrand M. (2019). Towards a Palliative Care Approach in Psychiatry—Do We Need a New Definition?. *Journal of Medical Ethics*.

[26] Strand M., Sjöstrand M., Lindblad A. (2020). A Palliative Care Approach in Psychiatry-Clinical Implication. *BMC Medical Ethics*.

[27] Westermair A. L., Buchman D. Z., Levitt S., Perrar K. M., Trachsel M. (2022). Palliative Psychiatry in a Narrow and in a Broad Sense: A Concept Clarification. *Australian and New Zealand Journal of Psychiatry*.

[28] Stoll J., Mathew A., Venkateswaran C., Prabhakaran A., Westermair A. L., Trachsel M. (2022). Palliative Psychiatry for Patients With Severe and Persistent Mental Illness: A Survey on the Attitudes of Psychiatrists in India Compared to Psychiatrists in Switzerland. *Frontiers in Psychiatry*.

[29] Trachsel M., Hodel M. A., Irwin S. A., Hoff P., Biller-Andorno N., Riese F. (2019). Acceptability of Palliative Care Approaches for Patients With Severe and Persistent Mental Illness: A Survey of Psychiatrists in Switzerland. *BMC Psychiatry*.

[30] Elie D., Marino A., Torres-Platas S. G. (2018). End-of-Life Care Preferences in Patients With Severe and Persistent Mental Illness and Chronic Medical Conditions—A Comparative Cross-Sectional Study. *American Journal of Geriatric Psychiatry*.

[31] Coulter A., Schuermeyer I., Sola C. (2021). Evaluating Ineffective Treatments: A Proposed Model for Discussing Futility in Psychiatric Illness. *Harvard Review of Psychiatry*.

[32] Geppert C. M. (2015). Futility in Chronic Anorexia Nervosa: A Concept Whose Time Has Not yet Come. *The American Journal of Bioethics*.

[33] Levitt S., Buchman D. Z. (2020). Applying Futility in Psychiatry: As Concept Whose Time Has Come. *Journal of Medical Ethics*.

[34] Eneslätt M., Helgesson G., Tishelman C. (2020). Exploring Community-Dwelling Older Adults’ Considerations About Values and Preferences for Future End-Of-Life Care: A Study From Sweden. *The Gerontologist*.

[35] Allard P., Gustafson L., Karlsson I., Björkstén K. S. (2009). Geriatric Psychiatry in Sweden Must Be Developed--Not Dismantled. New Investigation Shows Depressing Results. *Lakartidningen*.

[36] Weiner S. J., Wang S., Kelly B., Sharma G., Schwartz A. (2020). How Accurate Is the Medical Record? A Comparison of the Physician’s Note With a Concealed Audio Recording in Unannounced Standardized Patient Encounters. *Journal of the American Medical Informatics Association*.

